# Antigen-Induced Immunomodulation in the Pathogenesis of Atherosclerosis

**DOI:** 10.1155/2008/723539

**Published:** 2008-06-09

**Authors:** Natalia Milioti, Alexandra Bermudez-Fajardo, Manuel L. Penichet, Ernesto Oviedo-Orta

**Affiliations:** ^1^Faculty of Heath and Medical Sciences, University of Surrey, Guildford, Surrey GU2 7XH, UK; ^2^Department of Surgery, David Geffen School of Medicine, University of California, Los Angeles, CA 90095-6904, USA; ^3^Department of Microbiology, Immunology, and Molecular Genetics, David Geffen School of Medicine, University of California, Los Angeles, CA 90095 -1678, USA

## Abstract

Atherosclerosis is a chronic inflammatory disorder characterised by the accumulation of monocytes/macrophages, smooth muscle cells, and lymphocytes within the arterial wall in response to the release of proinflammatory molecules. Such accumulation results in the formation of the atherosclerotic plaque, which would eventually evolve to complications such as total artery occlusion, rupture, calcification, or aneurysm. Although the molecular mechanism responsible for the development of atherosclerosis is not completely understood, it is clear that the immune system plays a key role in the development of the atherosclerotic plaque and in its complications. There are multiple antigenic stimuli that have been associated with the pathogenesis of atherosclerosis. Most of these stimuli come from modified self-molecules such as oxidised low-density lipoproteins (oxLDLs), beta2glycoprotein1 (*β*2GP1), lipoprotein a (LP(a)), heat shock proteins (HSPs), and protein components of the extracellular matrix such as collagen and fibrinogen in the form of advanced glycation-end (AGE) products. In addition, several foreign antigens including bacteria such as Porphyromonas gingivalis and Chlamydia pneumoniae and viruses such as enterovirus and cytomegalovirus have been associated with atherosclerosis as potentially causative or bystander participants, adding another level of complexity to the analysis of the pathophysiology of atherosclerosis. The present review summarises the most important scientific findings published within the last two decades on the importance of antigens, antigen stimulation, and adaptive immune responses in the development of atherosclerotic plaques.

## 1. ATHEROSCLEROTIC PLAQUE FORMATION

**Figure 1:**
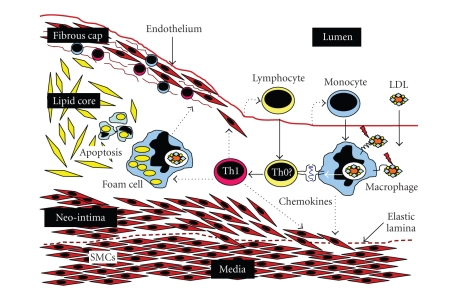
Inflammatory basis of atherosclerotic plaque formation. Led by inflammatory signals derived from the damaged endothelium, monocytes and lymphocytes migrate into the vessel wall. Monocytes differentiate into macrophages that recognise and phagocytiose oxidised LDL particles. The protein component of the LDL particle is processed and presented in the form of peptides by macrophages (also by dendritic cells) to T-lymphocytes in the context of the major histocompatibility complex class II (MHC-II). Other self or foreign antigens that may gain access to the vascular wall can also trigger similar mechanisms. It is believed that most of these lymphocytes differentiate in situ, under the influence of the specific antigen stimulation, into effector T-cells, but this has yet to be demonstrated. Upon activation, both macrophages and lymphocytes release a range of proinflammatory molecules including chemokines which stimulate the migration of smooth muscle cells (SMCs) from the media. SMCs contribute to foam cell and fibrous cap formation. This process is facilitated by cytokines such as IFN*γ* and TNF*α* secreted by proatherogenic Th1 cells and also IL-12 secreted by macrophages and foam cells. Eventually foam cells die by apoptosis in situ leaving nondegradable cholesterol crystals that form the lipid core of the plaque.

It is difficult to identify the factors responsible for the initiation of the atheroma lesion and/or the order in which these factors contribute to plaque formation. Nevertheless, it is known that endothelial dysfunction and high levels of circulating cholesterol, as oxLDL, play a key role in the proinflammatory process that triggers the first steps in the development of atherosclerotic plaques [1, 2]. Whatever the cause, these steps are characterised by an initially reversible accumulation of lipid-laden macrophages in the subendothelial space as a consequence of the increasing migration of blood-derived monocytes. These cells accumulate at focal points within the vascular wall of medium and small size arteries driven by chemokines and adhesion molecules produced by the damaged endothelium [3–5]. Monocytes differentiate in situ into macrophages which express membrane receptors such as Toll-like receptors and scavenger receptors that participate in the clearance of oxLDL [6, 7]. Lymphocytes can also transmigrate and accumulate within the arterial wall from the very earliest stages (Figure 1) [8].

**Figure 2:**
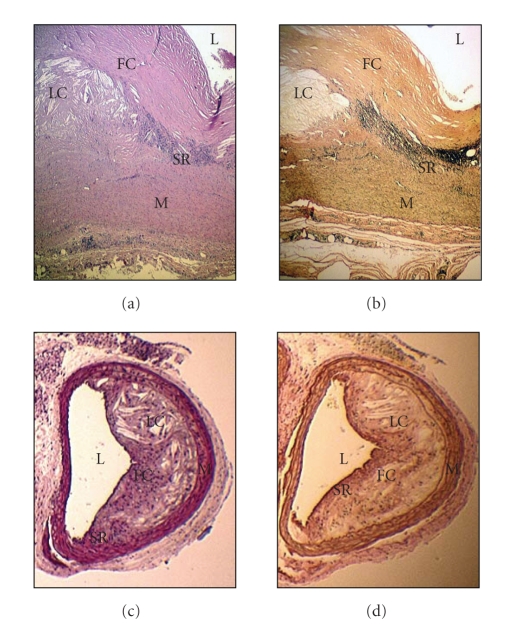
Morphological features of advanced atherosclerotic plaques. (a) and (b) show sections from a human carotid artery; (c) and (d) are sections from an apoE deficient mouse brachiocephalic artery. Sections (a) and (c) have been stained with haematoxylin and eosin and sections (b) and (d) with van Gieson staining (used to demonstrate the increase of collagen deposition and development of elastic fibres, a characteristic feature of the atherogenic process. A positive staining is depicted by a brown colour). L: *lumen* of the vessel; SR: *shoulder region* (it is believed to contain large numbers of proinflammatory cells including macrophages and lymphocytes, and it is the site related with the onset of the development of the atherosclerotic plaque); FC: *fibrous cap* (It also contains large numbers of mononuclear infiltrate and smooth muscle cells that have migrated from the *media* layer (M) and proliferated in response to the local inflammatory stimuli. It is also characterised by high collagen deposition and little or no endothelial cells); LC: lipid core (it contains mainly macrophage and smooth muscle cell-derived foam cells, apoptotic cells, and cholesterol crystals. Older lesions may also display signs of calcification). Contrasting differences can be recognised in the anatomic development of atherosclerotic plaques between human and mouse including the hypertrophy associated with the proliferation of the smooth muscle cells in the media layer and the fibrous cap. In humans, some lesions may also contain signs of intraplaque haemorrhage. Signs of plaque rupture are usually best recognised in mouse (reviewed in [13, 14]).

As the inflammatory process becomes chronic, smooth muscle cells also start to migrate from the media into the intima layer of the vessel, in response to chemokines and aided by the release of membrane metalloproteinases (MMPs) that enable them to break through the elastic lamina into the subendothelial space (Figure 1) [9, 10]. Persistence of inflammation creates a vicious circle of cell migration, dedifferentiation of smooth muscle cells, production of chemotactic and proinflammatory mediators, and cell death leading to vascular wall remodelling and formation of a new layer called neointima (Figures 1 and 2) [11, 12].

Neointima formation is a complex phenomenon that occurs in response to vessel wall damage in which repair and injury mechanisms give birth to areas rich in proinflammatory cells and collagen deposition [11, 12]. It is within these areas where a close contact between macrophages, dendritic cells (DCs), and lymphocytes has been reported. It has been speculated that the interaction between lymphocytes and DCs within the neointima is responsible for the development of local immune responses against exogenous and endogenous atherogenic antigens. These immune responses may contribute to cell death by apoptosis and accumulation of nondegradable cholesterol, contributing to the formation of the lipid core of the atherosclerotic plaque [15–18]. However, the precise mechanisms involved are not yet clear.

## 2. THE IMMUNOLOGIC NATURE OF THE ATHEROGENIC PROCESS

Atherosclerosis belongs to the group of chronic inflammatory diseases in which the cellular components of the immune system play a key role in its development and complications. The chronic accumulation of monocytes/macrophages, smooth muscle cells, and T-lymphocytes in response to the accumulation and release of proinflammatory molecules within the arterial wall constitutes the hallmark of a developing atherosclerotic plaque [15]. Although most of the antigenic stimuli that occur within atherosclerotic plaques come from modified self-molecules, the immune response triggered is remarkably similar to inflammatory reactions mounted against microbial organisms [19, 20]. The list of atherosclerosis-related antigens range from oxidised low-density lipoproteins (oxLDL), heat shock proteins (HSP) to protein components of the extracellular matrix such as collagen and fibrinogen (Table 1). On the other hand, the disputed role of foreign antigens, such as viruses and bacteria, in atherogenesis as causative or bystander participants in its development introduces another level of complexity to the analysis. This review summarises the most important scientific findings published within the last 2 decades on the importance of antigens, antigen stimulation, and adaptive immune responses in the development of atherosclerotic plaques.

## 3. ATHEROSCLEROSIS-RELATED ANTIGENS

As stated above, both endogenous and exogenous antigens are involved in atherogenesis. Table 1 summarises some of the most studied antigens in relation to atherosclerotic plaque formation.

### 3.1. Oxidised low-density lipoprotein (oxLDL)

**Figure 3:**
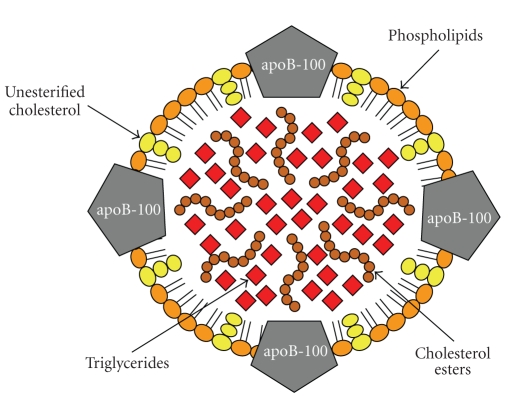
Schematic representation of the low-density lipoprotein particle (LDL). The LDL particle has a size of approximately 21–24 nm and is the main transporter of unesterified cholesterol, cholesterol esters, and triglycerides in the blood. It contains an outer layer composed of phospholipids and unesterified cholesterol in which a single protein is embedded, the apolipoprotein B-100 (apoB-100). These components are more susceptible to oxidation by free radicals in the subendothelial space during inflammation. They are also targets for the recognition of the LDL by scavenger receptors, proteoglycans, and low-density lipoprotein receptor (LDLr). The core of the particle contains primarily cholesterol esters and triglycerides. In atherogenesis, a large number of IgM antibodies are created in response to oxidative stress-modified phospholipids, whereas IgG antibodies and T-cell clones are generated against apoB-100.

OxLDL remains one of the most studied antigens in atherogenesis (Figure 3). LDL does not normally trigger immune responses in its native state; however when it gets oxidised, it can initiate inflammation-leading to atherosclerosis. OxLDL is considered a *neoantigen*, a term used to describe modified self-antigens for which normally the immune system is tolerant but which upon modification have the potential to excerpt autoreactive immune responses. Modified lipoprotein particles have the capacity to interact with the endothelium, triggering their accumulation and retention in the subendothelial space. The retained oxLDL particles induce macrophage foam cell formation, smooth muscle cell migration and proliferation [80] and, in addition, stimulate the secretion of inflammatory cytokines. OxLDL also induces the expression of adhesion molecules promoting endothelial cell dysfunction and leukocyte extravasation [81].

In atherosclerosis, increased levels of oxLDL have been found in the serum of patients admitted with acute myocardial infarction, and immunohistochemical studies revealed the presence of oxLDL within atherosclerotic plaques [82–85]. Proteoglycans, contained within the extracellular matrix of the arterial wall, interact with oxLDL via their carbohydrate groups, glycosamynoglycans (GAGs) as it has been described in vitro and in immunohistochemical studies using defective LDL unable to bind to GAGs [86]. The initial interaction of LDL with the proteoglycans leads to structural changes that expose the GAG-binding sites contributing to LDL retention. This interaction is mediated by apolipoprotein B (ApoB), which is the main protein component of LDL particles [87]. At least two proteoglycan-binding sequences within ApoB have been identified and shown to be necessary for retaining LDL particles within the intimal layer [80]. The interaction between the ApoB and the GAGs also facilitates LDL oxidation by oxygen-reactive species released by vascular cells or as a result of enzymatic modifications driven by endothelial cells, smooth muscle cells, or macrophages present within the atherosclerotic plaques [88]. Cholesterol molecules also have a high affinity for GAGs and for the LDL receptor. The accumulation of oxLDL within atherosclerotic plaques has been demonstrated, among other methods, by using monoclonal antibodies such as DLH3 that binds specifically to oxLDL but non to native LDL (nLDL) [89]. DLH3 binds to the oxidised phosphatidylcholine groups (oxPC) and to the phosphatidylcholine (PC)-apoB adducts formed when LDL is oxidised. PC-apoB adducts are considered to be the principal antibody targeted epitopes on the surface of the particles. Furthermore, oxPC also promote endothelial-monocyte adhesion by inducing the secretion of monocyte chemoattractant protein 1 (MCP-1) by endothelial cells [23], and can also be recognised by scavenger receptors such as CD36 which are involved in the oxLDL uptake by macrophages [90–93].

The initial interaction of proteoglycans with the LDL within the arterial wall leads lipid modifications that expose GAG binding sites. Cholesterol, cholesteryl esters and phospholipids are the main targets for lipid modifications and their role in the development of the disease has been extensively evaluated. These modifications play an important role, as stated above, in LDL retention within the arterial wall which is also facilitated by interactions with surface molecules such as apoE and lipoprotein lipase expressed on macrophages [94].

However, most studies have focused on the role of oxidised phospholipids (oxPLs) [95]. Peroxidation of the phospholipids starts in the fatty acids. The decomposition of fatty acids generates a spectrum of reactive species such as malondialdehyde (MDA), 4-hydroxynonenal, and the 1-palmitoyl-2-(5-oxovaleroyl)-glycero-3-phosphocholine molecule (POVPC) that can oxidise apolipoprotein B (ApoB) as well as other lipids, resulting in ox-PLs and oxidised protein-lipid adducts [96, 97].

It has also been established that oxLDL acts as a chemoattractant for monocytes allowing them to infiltrate the lesion site. OxLDL has been shown to induce monocyte adhesion to the endothelium by upregulating the expression of adhesion molecules on their surface, by inducing macrophage major histocompatibility class II (MHC-II) & LeuM3 cell surface expression and by accelerating monocyte differentiation in to macrophages [22]. Endothelial cells are also capable of oxidising LDL, contributing to a continuous generation of oxLDL within the lesion site, and to attract more monocytes. Other inflammatory mediators such as interleukin 1*β* (IL-1*β*), tumor necrosis factor alpha (TNF-*α*) and monocyte colony stimulating factor (M-CSF) can induce the expression of the oxLDL receptor (oxLDL-R) on the surface of endothelial cells, thus further contributing to oxLDL accumulation [22, 98].

Scavenger receptors expressed by macrophages play a pivotal role in LDL accumulation; such receptors include CD36 (a membrane glycoprotein), CD68, CXCL16, the scavenger receptors A & B1 (SR-A & SR-B1), and the lectin-type oxidised low-density lipoprotein receptor 1 (LOX1) [98, 99]. It is believed that the high expression of the scavenger receptors on macrophages mediates lipid accumulation and foam cell formation [100]. Following proteolytic processing inside the cell, fragments of the oxidised-modified ApoB protein are displayed on the surface of macrophages bound on MHC-II molecules. This process also leads to the upregulation of important molecules such as toll-like receptors TLR2 and TLR4 that induce proatherogenic immune responses [36]. There is also evidence that products of the inflammatory process such as endogenous HSP60 and LDL oxidation derivatives bind TLR4-CD14 complexes on monocytes and macrophages eliciting proinflammatory responses [30, 101, 102]. This has been associated with an enhanced production of cytokines, an enhancement of oxLDL uptake, and an increase adhesion of these cells to the endothelium mediated by IL-8 and NF*κ*B synthesis [101, 103].

The large size of the LDL molecule (2 × 10^6^ kDa) favours the exposure of many epitopes recognised by the antibodies generated, mainly IgM. OxLDL is known to be a very potent immunogen and the antibodies generated in response to its modifications are able to bind to many other similarly modified endogenous proteins [104]. It has been demonstrated that there is a molecular mimicry between the head of the PC groups of oxLDL and the PC groups expressed on the surface of many pathogens such as *Streptococcus pneumoniae* [105], which indicates that during an infection, more autoantibodies against oxLDL might be generated. Studies using experimental animal models have shown that epitopes generated during LDL oxidation, such as oxPC, are also generated on the surface of bacteria and on the surface of endothelial cells [21]. These epitopes bind to antibodies that will mediate removal of oxLDL and apoptotic cells [106]. Some of the oxidation-specific epitopes present on oxLDL are also presented on the surface of apoptotic cells in the lesion site, and play a role in the clearance of the damaged oxidised lipid molecules and of apoptotic cells generated during the inflammatory response within plaques [21].

### 3.2. Immunisation using oxLDL confers atheroprotection

A series of studies have shown the beneficial side of oxLDL. These studies have demonstrated that immune responses against this lipoprotein may protect against the development of the disease [107–111]. The first report came from Palinski et al. which immunised LDL receptor-deficient rabbits using homologous MDA-LDL. This treatment induced high titres of antibodies displaying equal specificity as those risen by the native particle and significantly reduced atherosclerotic plaque development [107]. Studies from other laboratories confirmed these results and showed that immunisation of hypercholesterolemic rabbits reduced T cell and oxLDL immunoreactivity within the neointima of immunized animals [108].

The effect of LDL immunisation on atheroprotection has also been assessed using mouse models of the disease. George et al. was the first to report the effect of MDA modified LDL immunisation in apoE-deficient mice (apoE^−/−^). Immunised mice developed high titres of anti-MDA-LDL antibodies and the treatment significantly reduced lesion size at the aortic sinus by more than half when compared with their control littermates immunised with PBS. However, they did not find differences between the groups with respect to cellular composition of the atherosclerotic plaques [109]. Later on, Freigang et al. showed that LDL receptor-deficient mice (LDLR^−/−^) immunised with homologous malondialdehyde-modified LDL (MDA-LDL) induced the synthesis of antibodies of different classes against distinctive epitopes on oxLDL and that this antibody response is significantly correlated with a reduction by approximately 40% of lesion size. However, they also showed that immunisation with MDA-LDL raised equivalent amounts of both T helper 1 (Th1)-related IgG2a and Th2-dependent IgG1 antibodies [110]. On the other hand, an elegant study carried out by Zhou et al. provided evidence of the involvement and control of the production of oxLDL-induced antibodies by T cells. They immunised apoE^−/−^ mice with homologous plaque homogenates or homologous MDA-LDL. They found that both antigen preparations reduced lesion development. The protective effect was associated with a specific raise of T-cell-dependent IgG antibodies against MDA-LDL and oxidised phospholipids which are correlated with the reduction in plaque size and circulating cholesterol levels [111]. Despite these demonstrations, the protective role of oxLDL during physiological conditions remains unknown and the immunological mechanisms related with it have not yet been fully studied.

### 3.3. *β*2-glycoprotein I (*β*2GpI)

Rheumatic patients suffering from the antiphospholipid syndrome produce large amounts of antiphospholipid antibodies. The standard phospholipid used to detect antiphospholipid antibodies is cardiolipin, which is prone to peroxidation and is also an important component of the oxLDL molecule [21]. A cofactor involved in anticardiolipin binding is the *β*2-glycoprotein I (*β*2GpI), a positively charged plasma protein circulating in the blood and also present in platelets and endothelial cells in atherosclerotic plaques [25]. Binding to the aPL antibodies requires a structural change in *β*2GpI which occurs when the protein binds to negatively charged phospholipids present in the atherosclerotic plaques. When transgenic animals are immunised with *β*2GpI, the atherosclerosis process is accelerated [26]. It has been reported that *β*2GpI can function as a scavenger receptor to mediate lipid engulfment by macrophages. Furthermore, histological studies showed that *β*2GpI is located in the subendothelial space in areas rich in CD4^+^ T cells [24]. A recent study showed that the adoptive transfer of *β*2GpI reactive T cells can promote the generation of fatty streaks in LDL^−/−^ mice, indicating that cellular autoimmunity is involved in the pathogenesis of atherosclerosis [25].

### 3.4. Lipoprotein(a) [Lp(a)]

Lp(a) is an antigen of relevance to atherosclerosis development [27]. Lp(a) is associated with apolipoprotein(a) (Apo-A), another glycoprotein. Lp(a) is present in the atherosclerotic plaques bound to fibrin. Furthermore, it may be internalised by macrophages within the plaques and induce the expression and secretion of chemoattractants from endothelial cells, thus triggering the attraction of monocytes in to atherosclerotic plaques. This effect is specifically attributed to the Apo-A component and suggests that in the presence of high levels of Apo-A, monocyte recruitment in to the vascular wall is favoured. The precise nature of the chemoattractant involved is not known yet, but GM-CSF and MCP-1 have already been discarded [27].

### 3.5. Lipoprotein-lipase (LPL)

A further self-antigen involved in lupus-related atherosclerosis is LPL [28]. It is a member of the lipase family that hydrolyses triglyceride molecules on lipoprotein molecules. LPL activity is significantly decreased with the progression of the disease due to the generation of anti-LPL antibodies [112, 113]. The hypothesis that has been formulated is that these antibodies might bind to LPL molecules on the surface of endothelial cells and obstruct lipid degradation by LPL, thus promoting lipid accumulation in the atherosclerotic plaques [113].

### 3.6. Advanced glycation end (AGE) products

A recent study suggests a possible role of AGE as facilitators of antigenic stimulation in atherosclerosis by promoting the maturation of dendritic cells (DCs) [29]. AGE products stimulate the upregulation of costimulatory and antigen presenting molecules on DCs which in turn causes T cell proliferation through the secretion of proinflammatory cytokines. This activation is mediated, at least in part by the upregulation of the receptors for AGE (RAGE) and the scavenger receptor A (SR-A), which is responsible for regulating cholesterol accumulation on DCs through the Jnk signaling pathway [29]. Immunohistological studies have confirmed the expression of AGE, as well as AGE receptors within atherosclerotic lesions. Cells expressing high levels of RAGE have been found located close to AGE, where normal RAGE is expressed in low levels in the endothelium [114, 115]. SR-A knock-out mice have decreased atherosclerotic lesions, therefore suggesting an indirect link between AGE stimulation and the development of atherosclerosis in these animals [116].

### 3.7. Heat shock proteins (HSPs)

HSPs are released from stressed endothelial cells and can act as chaperones in the process of denaturation of other proteins. They can induce the production of specific antibodies which usually accelerate atherosclerotic plaque development when used to immunise experimental animals [26]. Human and microbial HSP60 activate vascular endothelial cells and macrophages directly through CD14 and p38 mitogen-activated protein kinase signalling pathway in a similar manner as bacterial lipopolysaccharide (LPS) [30], leading to IL-6 and TNF-*α* secretion and promotion of atherosclerosis. HSPs are highly conserved among different species. Antibodies involved in the atherosclerotic development recognise both human and microbial HSPs [31].

### 3.8. Bacteria-derived antigens

The potential relationship between bacterial infections and the induction of atherosclerosis has been studied in different groups of cardiovascular patients including those who develop the disease but that lack the conventional risk factors associated with it such as hypercholesterolemia, high blood pressure, smoking and diabetes [51]. It has been speculated that bacterial infection may have a direct cytopathic effect on the vascular wall or that could act indirectly through the induction of an autoimmune inflammatory response involving mechanisms such as molecular mimicry and epitope spreading to generate atherosclerosis [19]. Several microbial components known to ligate pattern recognition receptors or heat shock proteins and unmethylated CpG DNA have been reported as ligands for toll-like receptors (TLRs), and therefore, have the potential to induce atherosclerosis [117]. TLRs are part of the sensing mechanisms in response to infections but it has been suggested that they may also play a contradictory role in inflammation leading to atherosclerosis. There is evidence showing that endothelial cells and macrophages in atherosclerotic lesions can upregulate TLR expression in response to microbial antigens [36]. It is known that autoantibodies such as those binding to endogenous human HSP60 and oxidised LDL can also activate TLR4 and induce proatherogenic immune responses. The response involves the secretion of proinflammatory cytokines, MMPs, and other inflammatory mediators (nitric oxide, endothelin-1) [118, 119].

An example is *Porphyromonas gingivalis* which has been detected within atherosclerotic plaques [37]. The inflammatory action of *P. gingivalis fimbriae* was shown to be mediated by ligation of TLR2, TLR4, CD14, and beta2-integrins and also by the upregulation of nuclear factor kappa-B (NF-*κ*B). It has also been observed that *P. gingivalis fimbriae* may promote atherosclerotic plaque rupture by inducing the secretion of MMPs [120–122]. Other pathogens studied in relation to atherosclerosis are *Bacteroides forsynthus,* where the protein A secreted by the bacterium acts through CD14 and TLR2 ligations to induce atherosclerosis, whereas in the case of *Streptococcus mutans* it is the protein AgI/II that acts through CD14 and TLR4 [37].

Similar mechanisms have been described for *Chlamydophila pneumoniae* (*C. pneumoniae*) infection where LPS and bacterial HSP act as ligands to TLRs. *C. pneumoniae* is an intracellular prokaryotic pathogen that infects humans provoking distinct forms of pneumonia, and it has been also proposed that it may cause chronic inflammatory diseases such as atherosclerosis [38]. Chlamydial LPS has been shown to induce macrophage foam cell formation and chlamydial HSP60 is known to contribute to LDL oxidation in the presence of macrophages on the lesion site. The presence of *C. pneumoniae* within atheroma lesions has been detected by PCR and immunohistochemistry. However, detection is sometimes difficult due to phases of activity and latency of the pathogen [123, 124]. The pathogen has been located within DCs in close proximity to T cells [125] but the precise mechanisms responsible for the induction of immune activation and atherosclerosis development remain to be clarified. There is evidence that patients with acute myocardial infarction have higher titres of antibodies against *C. pneumoniae* than control patients [126]. Moreover, *C. pneumoniae* has been also extracted and cultured from atherosclerotic plaques [125, 127, 128]. Experiments carried out in animal models demonstrated the induction of atherosclerosis by inoculation of *C. pneumoniae* [129]. *C. pneumoniae* can persistently infect epithelial cells and macrophages within human atherosclerotic plaques causing a chronic and nonlytic infection [128]. Immune responses against Chamydia spp. infection mainly involve CD4^+^ T-helper (Th)1 cells and antibodies, although other components such as CD8^+^ T cells also play a key role [130–132]. The relative contribution of these components to protection depends on several factors, such as the site of infection, whether it is a primary or secondary infection, and whether the infection is acute or persistent [133]. However, despite all the evidence supporting the role of Chlamydia infection in the development of atherosclerosis, its correlation with the development of complications remains controversial. A key element to the debate is the failure of recent human clinical trials and animal studies aiming to investigate the secondary preventive effect of antibiotics on atherosclerosis [128, 129, 134]. In these studies, antibiotic therapy was effective in clearing the acute infection, but failed to influence the atherogenic properties of *C. pneumoniae* unless the therapy was started early during the acute infection [134]. It has been hypothesised that this may be due to a sequestration of the organism within atherosclerotic plaques, that makes it inaccessible to both antibiotics and the cellular components of the immune response.

The microorganism *Helicobacter pylori—a* cause of gastrointestinal infections—has been also found to be present in atherosclerotic lesions but completely absent from healthy arteries [52, 53]. However, immunohistochemical studies could not detect its presence in the lesions but instead there was a strong cross-reactivity of the antibodies to the different elements of the plaque related to the acceleration of inflammatory events and plaque destabilisation.

Cross-reactivity has also been observed with antibodies against human HSP60 and *E. coli*-derived GroEL, an HSP [65]. The generation of anti-HSP antibodies can induce autoimmune reactions binding to HSP on endothelial cells at the lesion site where it is expressed at high levels due to shear stress triggered by blood pressure, stimulation by oxLDL in situ or by inducing the secretion of proinflammatory cytokines. The down stream effects are endothelial and macrophage damage and subsequent inflammatory events that lead to the pathogenesis of atherosclerosis [135–137].

### 3.9. Virus-derived antigens

Viruses have also been postulated as promoters of atherosclerosis. One of the most closely linked to this disease is cytomegalovirus. This virus infects the majority of the human population by targeting SMCs and endothelial cells producing a latent type of infection [138–142]. US28, one of the viral proteins expressed on the cell surface of the cytomegalovirus after infection, is a chemoattractant for SMCs. US28 and UL122 proteins were found to have an 11aa sequence homologous to human HSP60, and it is thought that antibodies against these viral proteins can bind to human HSP60 expressed on stressed endothelial cells [143]. The proteins also share some homology with nonstressed endothelial cell markers such as CD151, CD49f, and connexin 45 (Cx45). It is believed that during cytomegalovirus infection, antibodies generated against these proteins can bind, by molecular mimicry, to the surface markers on both nonstressed and already-stressed endothelial cells causing apoptosis of endothelial cells, which is considered to be one of the key early events in atherosclerotic plaque formation. It has been also suggested that endothelial cell stress induces HSP60 expression enhancing the binding of circulating autoantibodies and amplifying the endothelial cell damage [143]. Interesting results relating cytomegalovirus with atherosclerosis are derived from studies investigating the expression of the viperin gene. The human viperin gene has been suggested as a potential marker for cytomegalovirus infection [144]. Viperin, which is highly conserved among species, has a well-known antiviral effect and its use for the local treatment of cytomegalovirus has been recently proposed. Viperin is expressed by endothelial cells and SMCs in the vascular wall of disease vessels but no expression has been detected in the normal arteries [144].

Another pathogen associated with atherosclerosis is enterovirus, especially the enterovirus group coxsackie B virus [67]. High levels of enterovirus antibodies have been detected in patients with myocardial infraction but it has not yet been fully established whether the virus contributes to the pathogenesis of the disease [67, 68, 145]. Other infectious organisms that have been implicated in the pathogenesis of atherosclerosis involve a member of the herpes virus family that is known to induce atherosclerosis in chickens [146]. The virus alters cellular metabolism resulting in cholesterol accumulation which is a common mechanism proposed for all virally-induced atherosclerosis. There is also evidence that the virus promotes smooth muscle cells (SMCs) proliferation [147–150].

## 4. T-CELL ANTIGEN IMMUNE RESPONSES AND ATHEROSCLEROTIC PLAQUE DEVELOPMENT

T lymphocytes are present in atherosclerotic plaques at all stages of its development [15]. Most T cells within atherosclerotic plaques are CD4^+^ and a small fraction of the population consists of CD8^+^ T cells. CD4^+^ cells isolated from human plaques have been found to express the *α* *β* T cell receptor (*α* *β* TCR) [8, 151] that recognises antigens presented in the context of HLA-DR in the surface of APCs. The close proximity between T lymphocytes and APCs within atherosclerotic plaques supports the view that these lymphocytes are involved in antigen recognition and antigen-specific proliferation in the shoulder regions of atherosclerotic lesions [81]. They are attracted to the tissues by chemokines and adhesion molecules expressed on the surface of endothelial cells. Although the production of IgM, also called natural auto-antibodies, seems to be predominant in atherosclerosis, the presence in the serum of IgG antibodies specific to oxLDL epitopes is indicative of the involvement of CD4^+^ T cells in the process of affinity maturation and isotype class switching of B-cell clones specific to atherogenic antigens [21].

The unbalance between pro- and anti-inflammatory immune responses appears to be responsible for the development of atherosclerosis. The activation of naïve CD4^+^ T cells generates one of the two major types of functionally different effector T cells, the T-herper1 (Th1) or the Th2. The response of the former cells is considered to be proinflammatory in the context of atherosclerosis and is characterised by the secretion of IFN-*γ*, IL-12 and TNF-*α* which are all involved in macrophage activation. IFN-*γ* also drives Th1 cell differentiation that can be inhibited by IL-10 [21]. Th2 response is considered to be anti-inflammatory due to the secretion and action of IL-10 and other cytokines such as IL-4, IL-5, and IL-13, all linked to B cell activation and differentiation. Th2 differentiation is favoured by IL-4 and it can be inhibited by IFN-*γ*. Th1 is the predominant T cell subset found in atherosclerotic lesions [70, 152, 153].

The type of antibody produced, driven by Th1 or Th2 immune responses, also plays a key role in atherogenesis [21]. The synthesis of IgG1 antibodies indicates a predominant Th2 response while IgG2a is indicative of Th1 responses [21]. The regulation of the balance between Th1 and Th2 immune responses appears to be controlled by another T cell subset referred to as regulatory T cells (Tregs) (reviewed in [154]). It has been suggested that plaque size correlates with the number of Th1 cells present within the lesions [155].

On the other hand, Th2 activation and proliferation appears to be triggered by epitope-specific stimulation [7, 156–160] or by the induction of natural antibodies involved in the clearance of lipoprotein particles [95]. Auto-antibodies against oxLDL have been found circulating in the plasma. There is a correlation between the concentrations of these antibodies in plasma and lesion size [161]. Recent experimental evidence shows that pneumococcal vaccination using an animal model of atherosclerosis induces the production of anti-oxLDL IgM antibodies, which inversely correlates with the development of atherosclerotic plaques [162]. It has been also proposed that IgM antibodies may bind to oxLDL preventing its binding and degradation by macrophages, or even prevent the uptake of apoptotic cells by macrophages [163, 164]. However, the mechanisms involved in the production of these antibodies or their precise role in atherogenesis have not yet been addressed. Th2 responses are also recognised in advanced stages of atherosclerosis, when hypercholesterolemia is prominent and there seems to be a shift of the immune response towards a Th2 type, indicating that in late stages the immune system is trying to overcome the pro-inflammatory damage [152].

Noticeably, the therapeutic correction of the balance between these two types of responses has been pivotal for the development of novel interventions, such as vaccines against the development of the disease. Experiments carried out using inbred stains of mice show that C57BL/6 mice are more prone to develop Th1 responses and more atherosclerosis than BALB/c mice which are prone to develop Th2 responses and consequently atheroresistant [165]. It has also been noted that deletion of STAT6, a transcription factor required for the activation of Th2 responses, prone these mice to develop atherosclerosis [166]. Treatment of hypercholesteremic mice with recombinant IFN-*γ* also accelerates atherosclerotic plaque development [167], an effect that is reversed when mice receive the drug pentoxyfyllin, a potent Th1 blocker [155].

Switching the immune response to a Th2 type can be achieved by the expression of the anti-inflammatory cytokine IL-10 which suppresses the effect of proinflammatory cytokines such as IL-12 and IFN-*γ* [168, 169]. The athero-protective effect of IL-10 was noted even in mice fed a high-fat diet. However, the treatment failed to influence plasma cholesterol levels indicating that the IL-10 effects are due to modulation of the immune response involved in intraplaque inflammation mechanisms [169]. Deficiency of T-bet, a transcription factor required for Th1 differentiation, in experimental animals significantly reduced atherosclerotic lesions. This effect was linked to a reduction in number of proliferating smooth muscle cells in the intima layer [170]. T-bet deficient mice have also shown a skewed immune response towards the Th2-type when HSPs were administered to these mice [170]. These findings suggest that transcriptional regulation in T cell differentiation can represent a good target to immunomodulate atherosclerosis.

Just recently the first report appeared on the possible role of Th17 cells in cardiovascular disease [171]. Th17 cells are characterised by IL-17 (or IL-17A), IL-17F, IL-6, TNF-*α*, and IL-22 expressions. Their discovery has contributed to explain crucial regulatory mechanisms which until now the classic control by Th1 and Th2 or Treg cell-mediated mechanisms could not explain. Th17 cells have been suggested to play a key role in inflammation and autoimmunity. They have also been involved in the pathogenesis of hypersensitivity reactions. The study of their role in host defence mechanisms has just recently started and promises to be another area of high interest in cardiovascular biology research (see the following articles for a comprehensive review of the recently published literature on Th17 [172–177]). Cheng et al. have suggested that Th17/Treg balance may play a key role in controlling inflammation, plaque destabilization, and the onset of acute coronary syndrome. They investigated this hypothesis by assessing Th17/Treg functions through the analysis of T cell frequencies, secretion of specific cytokines, and production of key transcription factors in patients with acute myocardial infarction, unstable angina and stable angina. They found that Th17 cell numbers as well as its cytokines (IL-17, IL-6, and IL-23) and transcription factor (RORgammat) levels were significantly higher in patients with acute coronary syndrome as compared to controls. The study also showed a significant decrease in Treg number, Treg-related cytokines (IL-10 and TGF-*β*1), and Foxp3 levels in these patients as compared to stable angina and controls suggesting a potential role for Th17/Treg imbalance in plaque destabilization and the onset of ACS [171].

## 5. CONCLUSIONS

The multifactorial nature of atherosclerosis also applies to the number and quality of antigens capable of inducing proinflammatory/proatherogenic immune responses. The evidence accumulated so far supports the view that oxLDL is one of the most important atherogenic antigens, by virtue of being the main trigger of monocytes/macrophage and SMC infiltration, proliferation, and conversion in to foam cells in the neointima layer. The key role of other self-derived antigens such as HSPs and *β*2-GpI and the presence of circulating antibodies against them, that in most cases correlates with the clinical outcome, have been used to justify the classification of atherosclerosis as an inflammatory disease with an important autoimmune component. No less important is the role of foreign antigens derived, among others, from bacteria and viruses which might play a causal and/or at least a bystander effect contributing to the chronic inflammatory process and its complications. Finally, the role of T lymphocytes and the pro- and anti-inflammatory balances controlled by their different subsets has been shown to be crucial in the development of the disease. These responses are ultimately driven by the nature of the initial stimuli (the antigen) and supported by a complex cascade of events involving cytokines, components of the extracellular matrix, and even gene expression regulators such as transcription factors. Our current understanding of the immunopathogenic mechanisms involved in atherosclerotic plaque development has witnessed an enormous advance in the last decade, and some of this knowledge constitutes the foundation for the design of the next generation of drugs to combat cardiovascular disease and reduce its devastating consequences for the benefit of mankind.

## Figures and Tables

**Table 1:** Antigens that have been reported to be involved in atherogenesis.

	Antigen	Reference
*Autoantigens*	Oxidised low-density lipoprotein (LDL)	[21–23]
	Beta2glycoprotein1 (beta2GP1)	[24–26]
	Lipoprotein a (LP(a))	[27]
	Lipoprotein-lipase (LPL)	[28]
	Advanced glycation-end products (AGE)	[29]
	Heat-shock proteins	[30, 31]
	Collagen	[32–34]
	Fibrinogen	[35]

*Microbial antigens*	*Porphyromonas gingivalis*	[36, 37]
	*Chlamydia pneumoniae*	[38–49]
	*Bacteroides forsynthus*	[36]
	*Streptococcus mutans*	[36, 37, 50]
	*Helicobacter pylori*	[51–64]
	*Echerichia coli*	[65, 66]
	Enterovirus	[67–69]
	Cytomegalovirus	[22, 70–78]
	Viperin	[79]

## References

[1] Shashkin P, Dragulev B, Ley K (2005). Macrophage differentiation to foam cells. *Current Pharmaceutical Design*.

[2] Shi W, Wang X, Shih DM, Laubach VE, Navab M, Lusis AJ (2002). Paradoxical reduction of fatty streak formation in mice lacking endothelial nitric oxide synthase. *Circulation*.

[3] Pan JH, Sukhova GK, Yang JT (2004). Macrophage migration inhibitory factor deficiency impairs atherosclerosis in low-density lipoprotein receptor-deficient mice. *Circulation*.

[4] Lucas AD, Greaves DR (2001). Atherosclerosis: role of chemokines and macrophages. *Expert Reviews in Molecular Medicine*.

[5] Fan J, Watanabe T (2003). Inflammatory reactions in the pathogenesis of atherosclerosis. *Journal of Atherosclerosis and Thrombosis*.

[6] Michelsen KS, Arditi M (2006). Toll-like receptor signaling and atherosclerosis. *Current Opinion in Hematology*.

[7] Stoll G, Bendszus M (2006). Inflammation and atherosclerosis: novel insights into plaque formation and destabilization. *Stroke*.

[8] Jonasson L, Holm J, Skalli O, Bondjers G, Hansson GK (1986). Regional accumulations of T cells, macrophages, and smooth muscle cells in the human atherosclerotic plaque. *Arteriosclerosis*.

[9] Yan Z-Q, Hansson GK (2007). Innate immunity, macrophage activation, and atherosclerosis. *Immunological Reviews*.

[10] Newby AC (2006). Matrix metalloproteinases regulate migration, proliferation, and death of vascular smooth muscle cells by degrading matrix and non-matrix substrates. *Cardiovascular Research*.

[11] Hoofnagle MH, Thomas JA, Wamhoff BR, Owens GK (2006). Origin of neointimal smooth muscle: we've come full circle. *Arteriosclerosis, Thrombosis, and Vascular Biology*.

[12] De Meyer GR, Bult H (1997). Mechanisms of neointima formation—lessons from experimental models. *Vascular Medicine*.

[13] Jackson CL, Bennett MR, Biessen EAL, Johnson JL, Krams R (2007). Assessment of unstable atherosclerosis in mice. *Arteriosclerosis, Thrombosis, and Vascular Biology*.

[14] Jackson CL (2007). Is there life after plaque rupture?. *Biochemical Society Transactions*.

[15] Hansson GK, Libby P (2006). The immune response in atherosclerosis: a double-edged sword. *Nature Reviews Immunology*.

[16] Pinderski LJ, Fischbein MP, Subbanagounder G (2002). Overexpression of interleukin-10 by activated T lymphocytes inhibits atherosclerosis in LDL receptor-deficient mice by altering lymphocyte and macrophage phenotypes. *Circulation Research*.

[17] Dansky HM, Charlton SA, Harper MM, Smith JD (1997). T and B lymphocytes play a minor role in atherosclerotic plaque formation in the apolipoprotein E-deficient mouse. *Proceedings of the National Academy of Sciences of the United States of America*.

[18] Esaki T, Hayashi T, Muto E, Yamada K, Kuzuya M, Iguchi A (1997). Expression of inducible nitric oxide synthase in T lymphocytes and macrophages of cholesterol-fed rabbits. *Atherosclerosis*.

[19] Ludewig B, Krebs P, Scandella E (2004). Immunopathogenesis of atherosclerosis. *Journal of Leukocyte Biology*.

[20] Xu Q (2003). Infections, heat shock proteins, and atherosclerosis. *Current Opinion in Cardiology*.

[21] Hörkkö S, Binder CJ, Shaw PX (2000). Immunological responses to oxidized LDL. *Free Radical Biology and Medicine*.

[22] Aikawa M, Libby P (2004). The vulnerable atherosclerotic plaque: pathogenesis and therapeutic approach. *Cardiovascular Pathology*.

[23] Leitinger N (2005). Oxidized phospholipids as triggers of inflammation in atherosclerosis. *Molecular Nutrition and Food Research*.

[24] George J, Shoenfeld Y, Harats D (1999). The involvement of *β*2-glycoprotein I (*β*2-GPI) in human and murine atherosclerosis. *Journal of Autoimmunity*.

[25] George J, Harats D, Gilburd B (2000). Adoptive transfer of *β*2-glycoprotein i-reactive lymphocytes enhances early atherosclerosis in LDL receptor-deficient mice. *Circulation*.

[26] Jara LJ, Medina G, Vera-Lastra O, Amigo M-C (2006). Accelerated atherosclerosis, immune response and autoimmune rheumatic diseases. *Autoimmunity Reviews*.

[27] Poon M, Zhang X, Dunsky KG, Taubman MB, Harpel PC (1997). Apolipoprotein(a) induces monocyte chemotactic activity in human vascular endothelial cells. *Circulation*.

[28] de Carvalho JF, Borba EF, Viana VST, Bueno C, Leon EP, Bonfá E (2004). Anti-lipoprotein lipase antibodies: a new player in the complex atherosclerotic process in systemic lupus erythematosus?. *Arthritis & Rheumatism*.

[29] Ge J, Jia Q, Liang C (2005). Advanced glycosylation end products might promote atherosclerosis through inducing the immune maturation of dendritic cells. *Arteriosclerosis, Thrombosis, and Vascular Biology*.

[30] Kol A, Lichtman AH, Finberg RW, Libby P, Kurt-Jones EA (2000). Cutting edge: heat shock protein (HSP) 60 activates the innate immune response: CD14 is an essential receptor for HSP60 activation of mononuclear cells. *Journal of Immunology*.

[31] Lamb DJ, El-Sankary W, Ferns GAA (2003). Molecular mimicry in atherosclerosis: a role for heat shock proteins in immunisation. *Atherosclerosis*.

[32] Sander GE, Giles TD (2002). Cardiovascular complications of collagen vascular disease. *Current Treatment Options in Cardiovascular Medicine*.

[33] Amento EP, Ehsani N, Palmer H, Libby P (1991). Cytokines and growth factors positively and negatively regulate interstitial collagen gene expression in human vascular smooth muscle cells. *Arteriosclerosis and Thrombosis*.

[34] Thomas AH, Edelman ER, Stultz CM (2007). Collagen fragments modulate innate immunity. *Experimental Biology and Medicine*.

[35] Temelkova-Kurktschiev T, Koehler C, Henkel E, Hanefeld M (2002). Leukocyte count and fibrinogen are associated with carotid and femoral intima-media thickness in a risk population for diabetes. *Cardiovascular Research*.

[36] Hajishengallis G, Sharma A, Russell MW, Genco RJ (2002). Interactions of oral pathogens with Toll-like receptors: possible role in atherosclerosis. *Annals of Periodontology*.

[37] Yumoto H, Chou H-H, Takahashi Y, Davey M, Gibson FC, Genco CA (2005). Sensitization of human aortic endothelial cells to lipopolysaccharide via regulation of Toll-like receptor 4 by bacterial fimbria-dependent invasion. *Infection and Immunity*.

[38] Byrne GI, Kalayoglu MV (1999). *Chlamydia pneumoniae* and atherosclerosis: links to the disease process. *American Heart Journal*.

[39] Jha HC, Vardhan H, Gupta R, Varma R, Prasad J, Mittal A (2007). Higher incidence of persistent chronic infection of *Chlamydia pneumoniae* among coronary artery disease patients in India is a cause of concern. *BMC Infectious Diseases*.

[40] Ciervo A, Mancini F, Cassone A (2007). Transcription, expression, localization and immunoreactivity of *Chlamydophila pneumoniae* Phospholipase D protein. *Microbial Pathogenesis*.

[41] Gagliardi RJ, Silveira DR, Caffaro RA, dos Santos VP, Caiaffa-Filho HH (2007). *Chlamydia pneumoniae* and symptomatic carotid atherosclerotic plaque: a prospective study. *Arquivos de Neuro-Psiquiatria*.

[42] Ezzahiri R, Stassen FRM, Kurvers HRM, Dolmans V, Kitslaar PJEHM, Bruggeman CA (2006). *Chlamydia pneumoniae* infections augment atherosclerotic lesion formation: a role for serum amyloid P. *APMIS*.

[43] Yoshida T, Koide N, Mori I, Ito H, Yokochi T (2006). *Chlamydia pneumoniae* infection enhances lectin-like oxidized low-density lipoprotein receptor (LOX-1) expression on human endothelial cells. *FEMS Microbiology Letters*.

[44] Yavuz MT, Yavuz O, Yazici M (2006). Interaction between *Chlamydia pneumoniae* seropositivity, inflammation and risk factors for atherosclerosis in patients with severe coronary stenosis. *Scandinavian Journal of Clinical and Laboratory Investigation*.

[45] Wang SS, Tondella MLC, Bajpai A (2007). Circulating *Chlamydia pneumoniae* DNA and advanced coronary artery disease. *International Journal of Cardiology*.

[46] Hauer AD, de Vos P, Peterse N (2006). Delivery of *Chlamydia pneumoniae* to the vessel wall aggravates atherosclerosis in LDLr^−/−^
mice. *Cardiovascular Research*.

[47] Poppert S, Schlaupitz K, Marre R (2006). *Chlamydia pneumoniae* in an ex vivo human artery culture model. *Atherosclerosis*.

[48] Nazzal D, Cantéro A-V, Therville N (2006). *Chlamydia pneumoniae* alters mildly oxidized low-density lipoprotein-induced cell death in human endothelial cells, leading to necrosis rather than apoptosis. *The Journal of Infectious Diseases*.

[49] Sessa R, Di Pietro M, Schiavoni G (2006). *Chlamydia pneumoniae* in asymptomatic carotid atherosclerosis. *International Journal of Immunopathology and Pharmacology*.

[50] Nakano K, Inaba H, Nomura R (2006). Detection of cariogenic *Streptococcus mutans* in extirpated heart valve and atheromatous plaque specimens. *Journal of Clinical Microbiology*.

[51] Franceschi F, Sepulveda AR, Gasbarrini A (2002). Cross-reactivity of anti-CagA antibodies with vascular wall antigens: possible pathogenic link between Helicobacter pylori infection and atherosclerosis. *Circulation*.

[52] Kowalski M (2001). Helicobacter pylori (H. pylori) infection in coronary artery disease: influence of H. pylori eradication on coronary artery lumen after percutaneous transluminal coronary angioplasty. The detection of H. pylori specific dna in human coronary atherosclerotic plaque. *Journal of Physiology and Pharmacology*.

[53] Farsak B, Yildirir A, Akyön Y (2000). Detection of *Chlamydia pneumoniae* and *Helicobacter pylori* DNA in human atherosclerotic plaques by PCR. *Journal of Clinical Microbiology*.

[54] Okada T, Ayada K, Usui S (2007). Antibodies against heat shock protein 60 derived from *Helicobacter pylori*: diagnostic implications in cardiovascular disease. *Journal of Autoimmunity*.

[55] Kilic A, Onguru O, Tugcu H, Kilic S, Guney C, Bilge Y (2006). Detection of cytomegalovirus and *Helicobacter pylori* DNA in arterial walls with grade III atherosclerosis by PCR. *Polish Journal of Microbiology*.

[56] Adiloglu AK, Ocal A, Can R, Duver H, Yavuz T, Aridogan BC (2005). Detection of *Helicobacter pylori* and *Chlamydia pneumoniae* DNA in human coronary arteries and evaluation of the results with serologic evidence of inflammation. *Saudi Medical Journal*.

[57] Park MH, Min JY, Koh SB (2006). *Helicobacter pylori* infection and the CD14 C(-260)T gene polymorphism in ischemic stroke. *Thrombosis Research*.

[58] Cassar K, Bachoo P, Ford I, McGee M, Greaves M, Brittenden J (2004). *Helicobacter pylori* seropositivity is associated with enhanced platelet activation in patients with intermittent claudication. *Journal of Vascular Surgery*.

[59] Mach F, Sukhova GK, Michetti M, Libby P, Michetti P (2002). Influence of *Helicobacter pylori* infection during atherogenesis in vivo in mice. *Circulation Research*.

[60] Markus HS, Risley P, Mendall MA, Steinmetz H, Sitzer M (2002). *Helicobacter pylori* infection, the cytotoxin gene A strain, and carotid artery intima-media thickness. *Journal of Cardiovascular Risk*.

[61] Rechciński T, Kasprzak JD, Chmiela M, Krzemińska-pakuła M, Rudnicka W (2002). Patients with unstable angina pectoris present increased humoral response against *Helicobacter pylori* in comparison with patients with aggravated dyspepsia. *Acta Microbiologica Polonica*.

[62] Folsom AR, Nieto FJ, Sorlie P, Chambless LE, Graham DY (1998). *Helicobacter pylori* seropositivity and coronary heart disease incidence. *Circulation*.

[63] Cammarota G, Pasceri V, Papa A (1998). *Helicobacter pylori* infection and ischaemic heart disease. *Italian Journal of Gastroenterology and Hepatology*.

[64] Ossei-Gerning N, Moayyedi P, Smith S (1997). *Helicobacter pylori* infection is related to atheroma in patients undergoing coronary angiography. *Cardiovascular Research*.

[65] Mayr M, Metzler B, Kiechl S (1999). Endothelial cytotoxicity mediated by serum antibodies to heat shock proteins of *Escherichia coli* and *Chlamydia pneumoniae*: immune reactions to heat shock proteins as a possible link between infection and atherosclerosis. *Circulation*.

[66] Ünlü A, Türközkan N, Cimen B, Karabicak U, Yaman H (2001). The effect of *Escherichia coli*-derived lipopolysaccharides on plasma levels of malondialdehyde and 3-nitrotyrosine. *Clinical Chemistry and Laboratory Medicine*.

[67] Roivainen M, Alfthan G, Jousilahti P, Kimpimäki M, Hovi T, Tuomilehto J (1998). Enterovirus infections as a possible risk factor for myocardial infarction. *Circulation*.

[68] Kwon TW, Kim DK, Ye JS (2004). Detection of enterovirus, cytomegalovirus, and *Chlamydia pneumoniae* in atheromas. *Journal of Microbiology*.

[69] Cainelli F, Vento S (2002). Infections and solid organ transplant rejection: a cause-and-effect relationship?. *The Lancet Infectious Diseases*.

[70] Frostegard J, Nilsson J, Haegerstrand A, Hamsten A, Wigzell H, Gidlund M (1990). Oxidized low density lipoprotein induces differentiation and adhesion of human monocytes and the monocytic cell line U937. *Proceedings of the National Academy of Sciences of the United States of America*.

[71] Gredmark S, Jonasson L, Van Gosliga D, Ernerudh J, Söderberg-Nauclér C (2007). Active cytomegalovirus replication in patients with coronary disease. *Scandinavian Cardiovascular Journal*.

[72] Grahame-Clarke C (2005). Human cytomegalovirus, endothelial function and atherosclerosis. *Herpes*.

[73] Vliegen I, Duijvestijn A, Grauls G, Herngreen S, Bruggeman C, Stassen F (2004). Cytomegalovirus infection aggravates atherogenesis in apoE knockout mice by both local and systemic immune activation. *Microbes and Infection*.

[74] Altannavch Ts, Roubalová K, Brož J, Hrubá D, Andĕl M (2003). Serological markers of *Chlamydia pneumoniae*, cytomegalovirus and *Helicobacter pylori* infection in diabetic and non-diabetic patients with unstable angina pectoris. *Central European Journal of Public Health*.

[75] Taylor-Robinson D, Thomas BJ (1998). Antibodies to cytomegalovirus or *Chlamydia pneumoniae* and coronary heart disease. *The Lancet*.

[76] Adam E, Melnick JL, DeBakey ME (1997). Cytomegalovirus infection and atherosclerosis. *Central European Journal of Public Health*.

[77] Zhou YF, Guetta E, Yu ZX, Finkel T, Epstein SE (1996). Human cytomegalovirus increases modified low density lipoprotein uptake and scavenger receptor mRNA expression in vascular smooth muscle cells. *Journal of Clinical Investigation*.

[78] Koskinen P, Lemstrøm K, Mattila S, Häyry P, Nieminen MS (1996). Cytomegalovirus infection associated accelerated heart allograft arteriosclerosis may impair the late function of the graft. *Clinical Transplantation*.

[79] Puskás LG, Tiszlavicz L, Rázga Zs, Torday LL, Krenács T, Papp Gy (2005). Detection of nanobacteria-like particles in human atherosclerotic plaques. *Acta Biologica Hungarica*.

[80] Olofsson S-O, Borèn J (2005). Apolipoprotein B: a clinically important apolipoprotein which assembles atherogenic lipoproteins and promotes the development of atherosclerosis. *Journal of Internal Medicine*.

[81] Groyer É, Caligiuri G, Laschet-Khallou J, Nicoletti A (2006). Immunological aspects of atherosclerosis. *La Presse Médicale*.

[82] Hammer A, Kager G, Dohr G, Rabl H, Ghassempur I, Jürgens G (1995). Generation, characterization, and histochemical application of monoclonal antibodies selectively recognizing oxidatively modified apoB-containing serum lipoproteins. *Arteriosclerosis, Thrombosis, and Vascular Biology*.

[83] Festa A, Kopp HP, Schernthaner G, Menzel EJ (1998). Autoantibodies to oxidised low density lipoproteins in IDDM are inversely related to metabolic control and microvascular complications. *Diabetologia*.

[84] Iuliano L, Signore A, Violi F (1997). Uptake of oxidized LDL by human atherosclerotic plaque. *Circulation*.

[85] Yla-Herttuala S (1998). Is oxidized low-density lipoprotein present in vivo?. *Current Opinion in Lipidology*.

[86] Olsson U, Östergren-Lundén G, Moses J (2001). Glycosaminoglycan-lipoprotein interaction. *Glycoconjugate Journal*.

[87] Olofsson S-O, Asp L, Boren J (1999). The assembly and secretion of apolipoprotein B-containing lipoproteins. *Current Opinion in Lipidology*.

[88] Steinberg D, Carew TE, Fielding C (1989). Lipoproteins and the pathogenesis of atherosclerosis. *Circulation*.

[89] Itabe H (2003). Oxidized low-density lipoproteins: what is understood and what remains to be clarified. *Biological and Pharmaceutical Bulletin*.

[90] Rahaman SO, Lennon DJ, Febbraio M, Podrez EA, Hazen SL, Silverstein RL (2006). A CD36-dependent signaling cascade is necessary for macrophage foam cell formation. *Cell Metabolism*.

[91] Lim H-J, Lee S, Lee K-S (2006). PPAR*γ* activation induces CD36 expression and stimulates foam cell like changes in rVSMCs. *Prostaglandins & Other Lipid Mediators*.

[92] Wintergerst ES, Jelk J, Asmis R (1998). Differential expression of CD14, CD36 and the LDL receptor on human monocyte-derived macrophages: a novel cell culture system to study macrophage differentiation and heterogeneity. *Histochemistry and Cell Biology*.

[93] Ruiz-Velasco N, Domínguez A, Vega MA (2004). Statins upregulate CD36 expression in human monocytes, an effect strengthened when combined with PPAR-*γ* ligands Putative contribution of Rho GTPases in statin-induced CD36 expression. *Biochemical Pharmacology*.

[94] Kaplan M, Aviram M (2001). Retention of oxidized LDL by extracellular matrix proteoglycans leads to its uptake by macrophages: an alternative approach to study lipoproteins cellular uptake. *Arteriosclerosis, Thrombosis, and Vascular Biology*.

[95] Binder CJ, Shaw PX, Chang M-K (2005). The role of natural antibodies in atherogenesis. *Journal of Lipid Research*.

[96] Holvoet P, Perez G, Zhao Z, Brouwers E, Bernar H, Collen D (1995). Malondialdehyde-modified low density lipoproteins in patients with atherosclerotic disease. *Journal of Clinical Investigation*.

[97] Lecomte E, Artur Y, Chancerelle Y (1993). Malondialdehyde adducts to, and fragmentation of, apolipoprotein B from human plasma. *Clinica Chimica Acta*.

[98] Choudhury RP, Lee JM, Greaves DR (2005). Mechanisms of disease: macrophage-derived foam cells emerging as therapeutic targets in atherosclerosis. *Nature Clinical Practice Cardiovascular Medicine*.

[99] Moore KJ, Freeman MW (2006). Scavenger receptors in atherosclerosis: beyond lipid uptake. *Arteriosclerosis, Thrombosis, and Vascular Biology*.

[100] Bobryshev YV (2006). Monocyte recruitment and foam cell formation in atherosclerosis. *Micron*.

[101] Miller YI, Viriyakosol S, Binder CJ, Feramisco JR, Kirkland TN, Witztum JL (2003). Minimally modified LDL binds to CD14, induces macrophage spreading via TLR4/MD-2, and inhibits phagocytosis of apoptotic cells. *Journal of Biological Chemistry*.

[102] Pasini AF, Anselmi M, Garbin U (2007). Enhanced levels of oxidized low-density lipoprotein prime monocytes to cytokine overproduction via upregulation of CD14 and Toll-like receptor 4 in unstable angina. *Arteriosclerosis, Thrombosis, and Vascular Biology*.

[103] Yang QW, Mou L, Lv F-L (2005). Role of Toll-like receptor 4/NF-*κ*B pathway in monocyte-endothelial adhesion induced by low shear stress and ox-LDL. *Biorheology*.

[104] Libby P (2006). Inflammation and cardiovascular disease mechanisms. *American Journal of Clinical Nutrition*.

[105] Caligiuri G, Khallou-Laschet J, Vandaele M (2007). Phosphorylcholine-targeting immunization reduces atherosclerosis. *Journal of the American College of Cardiology*.

[106] Shaw PX, Goodyear CS, Chang M-K, Witztum JL, Silverman GJ (2003). The autoreactivity of anti-phosphorylcholine antibodies for atherosclerosis-associated neo-antigens and apoptotic cells. *Journal of Immunology*.

[107] Palinski W, Miller E, Witztum JL (1995). Immunization of low density lipoprotein (LDL) receptor-deficient rabbits with homologous malondialdehyde-modified LDL reduces atherogenesis. *Proceedings of the National Academy of Sciences of the United States of America*.

[108] Nilsson J, Calara F, Regnstrom J (1997). Immunization with homologous oxidized low density lipoprotein reduces neointimal formation after balloon injury in hypercholesterolemic rabbits. *Journal of the American College of Cardiology*.

[109] George J, Afek A, Gilburd B (1998). Hyperimmunization of apo-E-deficient mice with homologous malondialdehyde low-density lipoprotein suppresses early atherogenesis. *Atherosclerosis*.

[110] Freigang S, Hörkkö S, Miller E, Witztum JL, Palinski W (1998). Immunization of LDL receptor-deficient mice with homologous malondialdehyde-modified and native LDL reduces progression of atherosclerosis by mechanisms other than induction of high titers of antibodies to oxidative neoepitopes. *Arteriosclerosis, Thrombosis, and Vascular Biology*.

[111] Zhou X, Caligiuri G, Hamsten A, Lefvert AK, Hansson GK (2001). LDL immunization induces T-cell-dependent antibody formation and protection against atherosclerosis. *Arteriosclerosis, Thrombosis, and Vascular Biology*.

[112] Reichlin M, Fesmire J, Quintero-Del-Rio AI, Wolfson-Reichlin M (2002). Autoantibodies to lipoprotein lipase and dyslipidemia in systemic lupus erythematosus. *Arthritis & Rheumatism*.

[113] Mamputu JC, Desfaits AC, Renier G (1997). Lipoprotein lipase enhances human monocyte adhesion to aortic endothelial cells. *Journal of Lipid Research*.

[114] Basta G, Schmidt AM, De Caterina R (2004). Advanced glycation end products and vascular inflammation: implications for accelerated atherosclerosis in diabetes. *Cardiovascular Research*.

[115] Sun M, Yokoyama M, Ishiwata T, Asano G (1998). Deposition of advanced glycation end products (AGE) and expression of the receptor for AGE in cardiovascular tissue of the diabetic rat. *International Journal of Experimental Pathology*.

[116] Miyazaki A, Nakayama H, Horiuchi S (2002). Scavenger receptors that recognize advanced glycation end products. *Trends in Cardiovascular Medicine*.

[117] Tlaskalová-Hogenová H, Štěpánková R, Hudcovic T (2004). Commensal bacteria (normal microflora), mucosal immunity and chronic inflammatory and autoimmune diseases. *Immunology Letters*.

[118] Hollestelle SCG, de Vries MR, van Keulen JK (2004). Toll-like receptor 4 is involved in outward arterial remodeling. *Circulation*.

[119] de Graaf R, Kloppenburg G, Kitslaar PJHM, Bruggeman CA, Stassen F (2006). Human heat shock protein 60 stimulates vascular smooth muscle cell proliferation through Toll-like receptors 2 and 4. *Microbes and Infection*.

[120] Triantafilou M, Gamper FGJ, Lepper PM (2007). Lipopolysaccharides from atherosclerosis-associated bacteria antagonize TLR4, induce formation of TLR2/1/CD36 complexes in lipid rafts and trigger TLR2-induced inflammatory responses in human vascular endothelial cells. *Cellular Microbiology*.

[121] Ford PJ, Gemmell E, Timms P, Chan A, Preston FM, Seymour GJ (2007). Anti-P. gingivalis response correlates with atherosclerosis. *Journal of Dental Research*.

[122] Söder B, Airila Månsson S, Söder P-Ö, Kari K, Meurman J (2006). Levels of matrix metalloproteinases-8 and -9 with simultaneous presence of periodontal pathogens in gingival crevicular fluid as well as matrix metalloproteinase-9 and cholesterol in blood. *Journal of Periodontal Research*.

[123] Ieven M (2001). *Chlamydia pneumoniae* and atherosclerosis. *Verhandelingen—Koninklijke Academie voor Geneeskunde van Belgie*.

[124] Kaklikkaya I, Kaklikkaya N, Buruk K (2006). Investigation of *Chlamydia pneumoniae* DNA, chlamydial lipopolisaccharide antigens, and Helicobacter pylori DNA in atherosclerotic plaques of patients with aortoiliac occlusive disease. *Cardiovascular Pathology*.

[125] Bobryshev YV, Cao W, Phoon MC (2004). Detection of *Chlamydophila pneumoniae* in dendritic cells in atherosclerotic lesions. *Atherosclerosis*.

[126] Belland RJ, Ouellette SP, Gieffers J, Byrne GI (2004). *Chlamydia pneumoniae* and atherosclerosis. *Cellular Microbiology*.

[127] Sessa R, Di Pietro M, Schiavoni G (2004). Detection of *Chlamydia pneumoniae* in atherosclerotic coronary arteries. *International Journal of Immunopathology and Pharmacology*.

[128] Mussa FF, Chai H, Wang X, Yao Q, Lumsden AB, Chen C (2006). *Chlamydia pneumoniae* and vascular disease: an update. *Journal of Vascular Surgery*.

[129] de Kruif MD, van Gorp ECM, Keller TT, Ossewaarde JM, Ten Cate H (2005). *Chlamydia pneumoniae* infections in mouse models: relevance for atherosclerosis research. *Cardiovascular Research*.

[130] Su H, Caldwell HD (1995). CD4^+^ T cells play a significant role in adoptive immunity to *Chlamydia trachomatis* infection of the mouse genital tract. *Infection and Immunity*.

[131] Morrison SG, Morrison RP (2001). Resolution of secondary *Chlamydia trachomatis* genital tract infection in immune mice with depletion of both CD4^+^ and CD8^+^ T cells. *Infection and Immunity*.

[132] Brunham RC, Rey-Ladino J (2005). Immunology of Chlamydia infection: implications for a *Chlamydia trachomatis* vaccine. *Nature Reviews Immunology*.

[133] Entrican G, Buxton D, Longbottom D (2001). Chlamydial infection in sheep: immune control versus fetal pathology. *Journal of the Royal Society of Medicine*.

[134] Kuppuswamy VC, Gupta S (2006). Antibiotic therapy for coronary heart disease: the myth and the reality. *Timely Topics in Medicine. Cardiovascular Diseases*.

[135] Riganò R, Profumo E, Buttari B (2007). Heat shock proteins and autoimmunity in patients with carotid atherosclerosis. *Annals of the New York Academy of Sciences*.

[136] Hauet-Broere F, Wieten L, Guichelaar T, Berlo S, van der Zee R, Van Eden W (2006). Heat shock proteins induce T cell regulation of chronic inflammation. *Annals of the Rheumatic Diseases*.

[137] Ford PJ, Gemmell E, Chan A (2006). Inflammation, heat shock proteins and periodontal pathogens in atherosclerosis: an immunohistologic study. *Oral Microbiology and Immunology*.

[138] Liu R, Moroi M, Yamamoto M (2006). Presence and severity of *Chlamydia pneumoniae* and cytomegalovirus infection in coronary plaques are associated with acute coronary syndromes. *International Heart Journal*.

[139] Virok D, Kis Z, Kari L (2006). *Chlamydophila pneumoniae* and human cytomegalovirus in atherosclerotic carotid plaques—combined presence and possible interactions. *Acta Microbiologica et Immunologica Hungarica*.

[140] Westphal M, Lautenschlager I, Backhaus C (2006). Cytomegalovirus and proliferative signals in the vascular wall of CABG patients. *Thoracic and Cardiovascular Surgeon*.

[141] Müller BT, Huber R, Henrich B (2005). *Chlamydia pneumoniae*, herpes simplex virus and cytomegalovirus in symptomatic and asymptomatic high-grade internal carotid artery stenosis. Does infection influence plaque stability?. *Vasa*.

[142] Sun Y, Pei W, Welte T, Wu Y, Ye S, Yang Y (2005). Cytomegalovirus infection is associated with elevated interleukin-10 in coronary artery disease. *Atherosclerosis*.

[143] Bason C, Corrocher R, Lunardi C (2003). Interaction of antibodies against cytomegalovirus with heat-shock protein 60 in pathogenesis of atherosclerosis. *The Lancet*.

[144] Olofsson PS, Jatta K, Wågsäter D (2005). The antiviral cytomegalovirus inducible gene 5/viperin is expressed in atherosclerosis and regulated by proinflammatory agents. *Arteriosclerosis, Thrombosis, and Vascular Biology*.

[145] Fairweather D, Frisancho-Kiss S, Rose NR (2005). Viruses as adjuvants for autoimmunity: evidence from Coxsackievirus-induced myocarditis. *Reviews in Medical Virology*.

[146] Melnick JL, Adam E, DeBakey ME (1996). Cytomegalovirus and atherosclerosis. *Archivum Immunologiae et Therapiae Experimentalis*.

[147] Ibrahim AI, Obeid MT, Jouma MJ (2005). Detection of herpes simplex virus, cytomegalovirus and Epstein-Barr virus DNA in atherosclerotic plaques and in unaffected bypass grafts. *Journal of Clinical Virology*.

[148] Scheglovitova ON, Romanov YA, Maksianina EV, Svintsitskaya VA, Pronin AG (2002). Herpes simplex type I virus infected human vascular endothelial cells induce the production of anti-viral and proinflammatory factors by peripheral blood leukocytes in vitro. *Russian Journal of Immunology*.

[149] Shi Y, Tokunaga O (2002). Herpesvirus (HSV-1, EBV and CMV) infections in atherosclerotic compared with non-atherosclerotic aortic tissue. *Pathology International*.

[150] Kotronias D, Kapranos N (2005). Herpes simplex virus as a determinant risk factor for coronary artery atherosclerosis and myocardial infarction. *In Vivo*.

[151] Stemme S, Faber B, Holm J, Wiklund O, Witztum JL, Hansson GK (1995). T lymphocytes from human atherosclerotic plaques recognize oxidized low density lipoprotein. *Proceedings of the National Academy of Sciences of the United States of America*.

[152] Nilsson J, Hansson GK, Shah PK (2005). Immunomodulation of atherosclerosis: implications for vaccine development. *Arteriosclerosis, Thrombosis, and Vascular Biology*.

[153] Szodoray P, Timar O, Veres K (2006). Th1/Th2 imbalance, measured by circulating and intracytoplasmic inflammatory cytokines—immunological alterations in acute coronary syndrome and stable coronary artery disease. *Scandinavian Journal of Immunology*.

[154] Mallat Z, Ait-Oufella H, Tedgui A (2007). Regulatory T-cell immunity in atherosclerosis. *Trends in Cardiovascular Medicine*.

[155] Laurat E, Poirier B, Tupin E (2001). In vivo downregulation of T helper cell 1 immune responses reduces atherogenesis in apolipoprotein E-knockout mice. *Circulation*.

[156] Fredrikson GN, Berglund G, Alm R, Nilsson J-Å, Shah PK, Nilsson J (2006). Identification of autoantibodies in human plasma recognizing an apoB-100 LDL receptor binding site peptide. *Journal of Lipid Research*.

[157] Frederikson GN, Andersson L, Söderberg I (2005). Atheroprotective immunization with MDA-modified apo B-100 peptide sequences is associated with activation of Th2 specific antibody expression. *Autoimmunity*.

[158] Nilsson J (2005). Regelating protective immunity in atherosclerosis. *Circulation Research*.

[159] Fredrikson GN, Hedblad B, Berglund G (2003). Identification of immune responses against aldehyde-modified peptide sequences in apoB associated with cardiovascular disease. *Arteriosclerosis, Thrombosis, and Vascular Biology*.

[160] Caligiuri G, Nicoletti A, Poirier B, Hansson GK (2002). Protective immunity against atherosclerosis carried by B cells of hypercholesterolemic mice. *Journal of Clinical Investigation*.

[161] Palinski W, Witztum JL (2000). Immune responses to oxidative neoepitopes on LDL and phospholipids modulate the development of atherosclerosis. *Journal of Internal Medicine*.

[162] Binder CJ, Hörkkö S, Dewan A (2003). Pneumococcal vaccination decreases atherosclerotic lesion formation: molecular mimicry between *Streptococcus pneumoniae* and oxidized LDL. *Nature Medicine*.

[163] Kobayashi K, Tada K, Itabe H (2007). Distinguished effects of antiphospholipid antibodies and anti-oxidized LDL antibodies on oxidized LDL uptake by macrophages. *Lupus*.

[164] Shaw PX, Hörkkö S, Chang M-K (2000). Natural antibodies with the T15 idiotype may act in atherosclerosis, apoptotic clearance, and protective immunity. *Journal of Clinical Investigation*.

[165] Paigen B, Morrow A, Brandon C, Mitchell D, Holmes P (1985). Variation in susceptibility to atherosclerosis among inbred strains of mice. *Atherosclerosis*.

[166] Huber SA, Sakkinen P, David C, Newell MK, Tracy RP (2001). T helper-cell phenotype regulates atherosclerosis in mice under conditions of mild hypercholesterolemia. *Circulation*.

[167] Whitman SC, Ravisankar P, Elam H, Daugherty A (2000). Exogenous interferon-*γ* enhances atherosclerosis in apolipoprotein E-/- mice. *American Journal of Pathology*.

[168] Liu Y, Li D, Chen J (2006). Inhibition of atherogenesis in LDLR knockout mice by systemic delivery of adeno-associated virus type 2-hIL-10. *Atherosclerosis*.

[169] Pinderski Oslund LJ, Hedrick CC, Olvera T (1999). Interleukin-10 blocks atherosclerotic events in vitro and in vivo. *Arteriosclerosis, Thrombosis, and Vascular Biology*.

[170] Buono C, Binder CJ, Stavrakis G, Witztum JL, Glimcher LH, Lichtman AH (2005). T-bet deficiency reduces atherosclerosis and alters plaque antigen-specific immune responses. *Proceedings of the National Academy of Sciences of the United States of America*.

[171] Cheng X, Yu X, Ding Y (2008). The Th17/Treg imbalance in patients with acute coronary syndrome. *Clinical Immunology*.

[172] Bi Y, Liu G, Yang R (2007). Th17 cell induction and immune regulatory effects. *Journal of Cellular Physiology*.

[173] Afzali B, Lombardi G, Lechler RI, Lord GM (2007). The role of T helper 17 (Th17) and regulatory T cells (Treg) in human organ transplantation and autoimmune disease. *Clinical & Experimental Immunology*.

[174] Stockinger B, Veldhoen M (2007). Differentiation and function of Th17 T cells. *Current Opinion in Immunology*.

[175] Schmidt-Weber CB, Akdis M, Akdis CA (2007). Th17 cells in the big picture of immunology. *Journal of Allergy and Clinical Immunology*.

[176] Bettelli E, Korn T, Kuchroo VK (2007). Th17: the third member of the effector T cell trilogy. *Current Opinion in Immunology*.

[177] Weaver CT, Harrington LE, Mangan PR, Gavrieli M, Murphy KM (2006). Th17: an effector CD4 T cell lineage with regulatory T cell ties. *Immunity*.

